# Color‐Changing Paints Enabled by Photoresponsive Combinations of Bio‐Inspired Colorants and Semiconductors

**DOI:** 10.1002/advs.202302652

**Published:** 2023-10-03

**Authors:** Cassandra L. Martin, Kaitlyn R. Flynn, Taehwan Kim, Skyler K. Nikolic, Leila F. Deravi, Daniel J. Wilson

**Affiliations:** ^1^ Kostas Research Institute at Northeastern University Burlington MA 01803 USA; ^2^ Department of Chemistry and Chemical Biology Northeastern University Boston MA 02115 USA; ^3^ Department of Chemical Engineering Northeastern University Boston MA 02115 USA

**Keywords:** coatings, colors, paints, photochromism, titanium dioxide

## Abstract

Modern paints and coatings are designed for a variety of applications, ranging from fine art to extraterrestrial thermal control. These systems can be engineered to provide lasting color, but there are a limited number of materials that can undergo transient changes in their visual appearance in response to external stimuli without requirements for advanced fabrication strategies. The authors describe color‐changing paint formulations that leverage the redox‐dependent absorption profile of xanthommatin, a small‐molecule colorant found throughout biology, and the electronic properties of titanium dioxide, a ubiquitous whitening agent in commercial coatings. This combination yields reversible photoreduction upon exposure to sunlight, shifting from the oxidized (yellow) form of xanthommatin, to the reduced (red) state. The extent of photoreduction is dependent on the loading density and size of titanium dioxide particles, generating changes in hue angle as large as 77% upon irradiation. These coatings can be blended with non‐responsive supplemental colorants to expand the accessible color palette, and irradiated through masks to create transient, disappearing artwork. These formulations demonstrate energy‐efficient photochromism using a simple combination of a redox‐active dye and metal oxide semiconductor, highlighting the utility of these materials for the development of optically dynamic light‐harvesting materials.

## Graphical abstract

Based on the color‐changing capabilities of the natural biochrome xanthommatin, the authors have developed photoresponsive coatings that undergo reversible hue shifts in response to sunlight. In these paints, redox activity of the functional color‐changing component is enabled by embedded titanium dioxide particles, highlighting the photochromic behavior of this unique material combination.

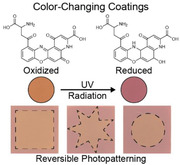

## 1 Introduction

For more than 100 000 years, natural materials have been incorporated into paints and coatings for decoration and protection.^[^ ^1^ ^]^ From rudimentary combinations of naturally available colorants including ochre,^[^ ^2^ ^]^ bone ash, calcite,^[^ ^3^ ^]^ charcoal,^[^ ^4^ ^]^ and lapis lazuli,^[^ ^5^ ^]^ blended into animal fat^[^ ^6^ ^]^ and oils^[^ ^7^ ^]^ to engineered coatings featuring synthetic pigments, paints are versatile tools for applications ranging from prolonging the service lifetime of buildings^[^ ^8^ ^]^ and infrastructure^[^ ^9^ ^]^ to design and self‐expression. In modern formulation science, precise combinations of natural and synthetic materials, including pigments, soluble dyes, dispersing agents, and supplemental additives (e.g., fillers, extenders, and texture control agents)^[^ ^10^ ^]^ are employed to achieve target color and gloss values in a wide variety of paint matrices (e.g., synthetic and natural resins, lacquers, thermosetting systems).^[^ ^11^ ^]^ A broad abundance of compatible ingredients enables the targeted design of paints with well‐controlled optical properties tailored toward application‐specific durability and performance requirements. These paints can typically maintain their original color and sheen over long time periods (e.g., 10 years)^[^ ^8^ ^]^ before re‐application is necessary.

While color fastness is an advantage in many commercial and industrial applications, a growing number of responsive materials have been developed to create coating formulations that can undergo changes in response to their surroundings or an applied stimulus. Broadly, these include electrochromic materials (e.g., PEDOT:PSS, 4,4′‐bipyridinium salts, tungsten oxide)^[^ ^12^ ^]^ that can provide dramatic changes in light transmittance in response to an applied voltage, films comprised of arrayed particles or other patterned features that undergo changes in size and structural coloration in response to vapor adsorption,^[^ ^13^ ^]^ ion deposition,^[^ ^14^ ^]^ or mechanical manipulation,^[^ ^15^ ^]^ and thermochromic or photochromic dyes that undergo reversible changes in their chemical structure (e.g., *cis‐trans* isomerization, ring opening/closure)^[^ ^16^ ^]^ upon exposure to heat or light, respectively. For each of these classes of materials, the source of input energy required to drive specific optical changes is an important consideration in the design of integrated and scalable technologies, where performance features (e.g., switching speed) and the accessible color palette are directly linked to color‐changing materials and their activation thresholds. For example, while electrochromic devices can change color within milliseconds,^[^ ^17^ ^]^ integration of control circuitry and multilayered device requires multi‐step fabrication strategies.^[^ ^18^ ^]^ Materials that provide dynamic structural coloration are resistant to photobleaching^[^ ^19^ ^]^ and can be tuned to provide broad color palettes but often must be directly accessible by controlling species in their surroundings, which may limit the durability of these systems over time in harsh or extreme environments. Both thermochromic and photochromic materials can be embedded or encapsulated in a durable bulk matrix, such as paint. While the leuco dyes^[^ ^20^ ^]^ and liquid crystal materials^[^ ^21^ ^]^ that enable thermochromic performance offer reproducible color switching, these formulations can require substantial energy from an outside source to function. In cases where localized heating (e.g., 25−80 °C)^[^ ^20^ ^]^ is not practical, ambient sources of energy, such as sunlight, can be a preferred strategy to enable responsive color change across a variety of environments.

Material combinations that leverage natural functional materials and mechanisms are among the most energy‐efficient formulations that can undergo optical and mechanical changes upon exposure to light, as they can be tuned to respond to the ambient energy of the sun. Examples of bio‐inspired photoresponsive materials include self‐cleaning membranes,^[^ ^22^ ^]^ actuators that bend toward light via photothermal^[^ ^23^ ^]^ and optomagentic^[^ ^24^ ^]^ mechanisms, and color‐changing hydrogel formulations.^[^ ^25^ ^]^ Interestingly, materials typically incorporated into paint and coating systems can catalyze alterations of light‐absorbing materials in the presence solar radiation. For example, titanium dioxide (TiO_2_), a ubiquitous pigment used to hide the underlying substrate and control brightness in nearly all commercially available paint formulations,^[^ ^26^ ^]^ can catalyze destruction of organic dyes in response to increasing doses of sunlight.^[^ ^27^ ^]^ This approach highlights opportunities to control natural colorants by leveraging the electronic properties of inorganic materials in solid‐state formulations. Recently, we reported that the color‐changing natural colorant xanthommatin (Xa), the primary pigment in the skin of arthropods and cephalopods,^[^ ^28^ ^]^ can undergo pH‐regulated color changes in response to sunlight to enable colorimetric measurements of solar exposure.^[^ ^29^ ^]^ The redox‐dependent color of Xa can also be controlled electrochemically,^[^ ^18b^ ^]^ indicating that transient changes in the optical properties of this natural colorant may be controlled using combinations of semiconductors and sunlight.

In this manuscript, we report the development of photochromic coating formulations that reversibly shift between the oxidized (yellow) and reduced (red) states of Xa in response to solar radiation. We incorporated Xa into a water‐based polyurethane paint matrix with TiO_2_, a known photocatalyst,^[^ ^30^ ^]^ of two different particle sizes to promote Xa reduction upon exposure to sunlight. We prepared our spray‐processible paint samples with different molar ratios of Xa to TiO_2_ and measured dose‐dependent color change, relaxation toward pre‐irradiation color in the absence of sunlight, and reversibility of light‐driven color change over multiple cycles of irradiation and relaxation. To more closely examine the spectral contributions that enable this observed effect, we exposed our samples to UVA, UVB, UVC, and visible/IR light individually and examined the intensity of resulting color changes. Additionally, we expanded the color palette of our coatings by blending combinations of Xa and TiO_2_ with non‐photoresponsive supplement colorants. Finally, we demonstrated that selective irradiation of geometric designs onto coated samples enables formation of complex, contrasting patterns that disappear over time.

## 2 Results and Discussion

### 2.1 Irradiation Experiments

In this work, we used Xa as a colorant in water‐based polyurethane coatings and explored its color changing abilities under sunlight when embedded in a solid paint matrix. To quantify color changes in our coatings, we measured hue angle before and after irradiation (Equation (1)). A color's hue is generally defined as how similar the color is to red, yellow, blue, green, or a combination of two of those colors.^[^ ^31^ ^]^ In the CIE Lab color space,^[^ ^32^ ^]^ hue angle is the angle between the *a* (red to green) and *b* (yellow to blue) axes. This value provided a strong indication as to how the overall color of our coatings changed in response to irradiation, as opposed to changes in single components of overall color (e.g., RGB color channels). Because pH regulates the color‐changing capabilities of Xa,^[^ ^33^ ^]^ we measured the pH of the water‐based polyurethane (Figure S1, Supporting Information) and found that it was between 7–8, indicating that the color changing capabilities of the Xa were not inhibited by the chemical environment of our coatings. When we exposed coatings consisting solely of Xa in the polyurethane matrix to 1100 W m^−2^ sunlight for 60 min, we observed hue angle changes of 2.7 ± 0.4% (63.5 ± 0.6° to 65.2 ± 0.4°), indicating almost no change from the initial color of the sample. The only observable change was a slight darkening of the coating. We also evaluated coatings prepared from the polyurethane matrix alone and saw minimal color change due to sunlight, indicating that the prominent hue shifts in Xa‐containing coatings were not due alterations in the polyurethane paint base (Table S1, Supporting Information).

We incorporated TiO_2_, a common photocatalyst, into our formulations to facilitate Xa reduction and color change in response to irradiation. We again tested the pH of the formulations and found that the addition of TiO_2_ led to no change in the pH of the mixture and would therefore not affect Xa's ability to change color (Figure S1, Supporting Information). We selected TiO_2_ as a component of our coatings to meet critical formulation design features, including requirements for controlled visible color and electrical activation of our dynamic optical ingredient, xanthommatin. TiO_2_ is highly effective at scattering visible light, and is a ubiquitous whitening agent used in color development across a wide variety of products (e.g., paints, cosmetics, and food).^[^ ^26^ ^]^ Due to its optical properties TiO_2_ is universally employed, typically as a mixture of anatase and rutile types,^[^ ^34^ ^]^ as a hiding pigment in the development of paints and coatings to provide opacity to thin films and hide the underlying substrate.^[^ ^35^ ^]^ Additionally, TiO_2_ particles can be used to create high surface area, porous semiconductor films that perform charge collection and electron conduction in photovoltaic devices. When these films are functionalized with dyes, the structure and size features of the TiO_2_ particles enhance light harvesting by the sensitizing agent, facilitating injection of electrons into the solid layer.^[^ ^36^ ^]^ The electronic features of TiO_2_ paired with the light‐responsive and electrochromic properties of Xa^[^ ^29^, ^37^ ^]^ give rise to reversible color change of Xa in our coatings, enabled by direct electronic communication between the semiconductor and redox‐active colorant in these formulations.

We used two different sizes of TiO_2_ particles—146.5 ± 43.9 nm and 24.2 ± 5.0 nm in diameter—and hypothesized that decreasing the particle size would increase the accessible surface area of the TiO_2_ in our formulations, providing more surface area for photoreduction and stronger subsequent color changes (Figures S2 and S3, Supporting Information).^[^ ^38^ ^]^ We calculated the approximate amount of TiO_2_ surface area in each of our formulations (Figure S4, Supporting Information) which revealed that coating formulations with smaller TiO_2_ particles offered ≈7.5 times the amount of TiO_2_ surface than equivalent formulations made with the larger particles. We expected that this difference would dictate the extent of color change in our coatings upon irradiation.

**Figure 1:**
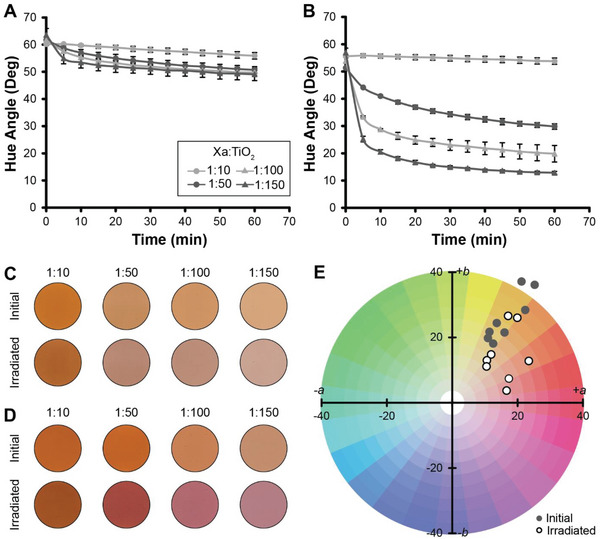
Photoreduction of Xa‐based coatings in sunlight. Changes in hue angle for coatings containing Xa and A) larger TiO_2_ particles and B) smaller TiO_2_ particles over 60 min of irradiation. Results depict an average of three formulation replicates and error is reported as standard deviation. Representative images of color‐changing coatings prepared using C) larger TiO_2_ particle and D) smaller TiO_2_ particles. Images represent ≈400 mm^2^ of coating surface. E) CIE Lab color coordinate diagram depicting chromaticity values of Xa‐based coatings before and after irradiation. The color coordinate diagram in Figure S5 (Supporting Information) shows connections (lines) between the initial coating color and its corresponding color after irradiation.

We prepared samples with 1:10, 1:50, 1:100, and 1:150 molar ratios of Xa to TiO_2_ for both particle sizes and irradiated the samples using simulated solar light, imaging the samples every 5 min for 60 min to record color change (**Figure** **1**). For the 1:10 sample with both larger (Figure 1A) and smaller (Figure 1B) TiO_2_ particles, we observed 4.3 ± 1.2° and 1.8 ± 0.6° changes in hue angle, respectively. Compared to the other conditions, these changes were small and may indicate that there was not enough accessible TiO_2_ to fully catalyze Xa reduction (Figure 1C,D). Interestingly, the samples containing larger TiO_2_ particles had a slightly greater hue angle change than those containing smaller TiO_2_ particles. This may be because the accessible total surface areas of both the larger and smaller TiO_2_ in the 1:10 formulations are more similar to each other than in formulations with higher TiO_2_ loading densities (Figure S4, Supporting Information). In addition, the larger TiO_2_ particles have a greater brightness (*L* value) than the smaller TiO_2_ particles, creating a different white base (Table S2, Supporting Information), which could lead to differences in the visible color between the two samples that cannot be attributed to the quantities of colorants alone. This variation may influence the magnitude of the visible color change in these coatings after solar irradiation.

In the other samples prepared with larger TiO_2_ particles, we observed differences in hue angle of 11.0 ± 1.5°, 11.8 ± 1.8°, and 14.5 ± 0.4° for the 1:50, 1:100, and 1:150 samples, respectively. An obvious shift from yellow to red indicated that the Xa in the coatings was reduced (Figure 1A,C). However, these results showed that there was not a steady change in hue angle over the full 60 min period (Figure 1A). The 1:50, 1:100, and 1:150 samples reached 85% of the total hue angle change in 39.3 ± 4.8, 32.9 ± 2.2, and 27.0 ± 4.7 min, respectively. Although the average values of these results demonstrated a direct correlation between TiO_2_ loading density and the rate of Xa photoreduction, the only statistically different times required to reach the 85% threshold are between the 1:50 and 1:150 samples. We suspect that this may be because the difference in accessible TiO_2_ surface area between the 1:50 and 1:150 samples is high enough to promote detectable differences in the rate of Xa photoreduction—the surface area of the 1:150 sample is approximately three times that of the 1:50 sample (Figure S4, Supporting Information). The differences in TiO_2_ surface area between the 1:50 and 1:100 (1:2) and 1:100 and 1:150 (2:3) samples are smaller, which may explain why the differences in the rate of photoreduction are not statistically significant.

Color change after irradiation was even more prominent in the 1:50, 1:100, and 1:150 samples with smaller TiO_2_ particles (Figure 1B,D). In each case, there was a significant decrease in hue angle after only 5 min of irradiation. For the 1:150 sample, over 50% of the hue angle change occurred in the first 5 min of irradiation. The differences in hue angle before and after irradiation were 21.5 ± 1.5°, 34.0 ± 3.3°, and 44.1 ± 1.8° for the 1:50, 1:100, and 1:150 samples, respectively. These results supported our hypothesis that increasing the surface area of available TiO_2_ in the coating would lead to enhanced photoreduction of Xa and strong color change. For example, we observed a 22.8 ± 0.9% change in hue angle for the 1:150 sample with larger TiO_2_ particles, and a 77.4 ± 1.2% change in the 1:150 sample with smaller TiO_2_ particles. Smaller TiO_2_ particles provided a greater ultimate color change in hue angle. In samples prepared using these particles, the rate at which maximum color change was achieved within 60 min increased with increasing particle loading density. The 1:50, 1:100, and 1:150 samples with smaller TiO_2_ particles reached 85% of their final hue angle change in 36.5 ± 1.5, 20.5 ± 5.5, and 12.2 ± 0.5 min, respectively. These results illustrate a direct relationship between both the rate and overall amount of hue angle change and the particle loading density. In addition, unlike the samples with the larger TiO_2_ particles, the difference in the estimated surface area (Figure S4, Supporting Information) between the formulations with the smaller particles is greater, which leads to distinctly different rates of photoreduction between the samples. TiO_2_ acts as a whitening agent in the coatings, which allowed us to control the brightness of our formulations (Table S2, Supporting Information), resulting in a range of visible appearances before and after irradiation (Figure 1E). Furthermore, because samples prepared with smaller TiO_2_ particles achieved a greater change in hue angle compared to samples prepared with larger TiO_2_ particles, small particle formulations offer more resolved control over final coating color and a wider range of accessible colors within a single formulation.

In addition to the changes in the *a* and *b* values of the coatings upon irradiation, we also observed that there was a change in the *L* value, or lightness, of the samples. In all conditions, the *L* value decreased and became darker upon irradiation (Table S2, Supporting Information). While the red color of the reduced coatings is visually darker than their initial, oxidized form, resulting in an intuitive decrease in brightness, this change can also be attributed to differences in the extinction coefficient of the oxidized versus reduced forms of xanthommatin.^[^ ^39^ ^]^ From this, we can infer that the reduced form of Xa is a better light absorber than the oxidized form, which leads to the consistent decrease in lightness (*L* value) that we see for each condition upon irradiation.

We decided to further explore the effects of TiO_2_ loading density on the rate of photoreduction and overall change in hue angle upon irradiation by preparing coating formulations of 1:200 and 1:250 molar ratio of Xa to TiO_2_ with both larger and smaller TiO_2_ particles and irradiating them over 60 min (Figure S6, Supporting Information). For both the larger and smaller TiO_2_ samples, we did not observe a direct correlation between rate of photoreduction and TiO_2_ loading density. It took the 1:200 and 1:250 samples with the larger TiO_2_ particles 32.4 ± 14.2 and 39.8 ± 5.7 min to reach 85% of the total hue angle change, respectively. In addition, it took the 1:200 and 1:250 samples with the smaller TiO_2_ particles 19.3 ± 8.5 and 16.9 ± 6.4 min to achieve 85% of the total hue angle change. Although there were still distinct color changes in all of the coatings, it is also important to note that the variability in these measurements was greater for all the 1:200 and 1:250 samples compared to the 1:50, 1:100, and 1:150 samples, which may be attributed to inconsistencies in the homogeneity of these paint mixtures containing high quantities of solid particles. All of our samples were applied manually via airbrushing, which is a potential source of color inconsistency from sample to sample. Across all of the formulation conditions we evaluated, increasing the amount of TiO_2_ beyond the 1:150 formulation did not directly lead to faster Xa photoreduction.

### 2.2 Relaxation Experiments

**Figure 2:**
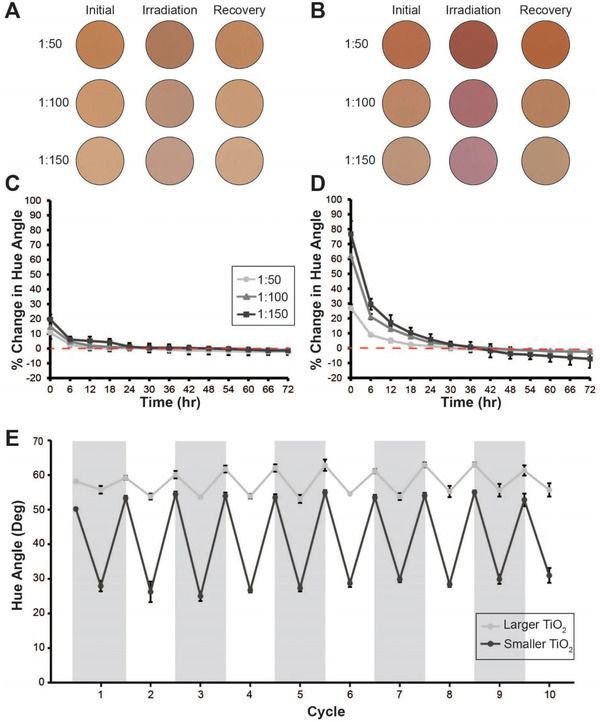
Reversibility of light‐driven color change. Representative images for coatings composed of A) larger and B) smaller TiO_2_ particles before irradiation, immediately after irradiation, and 72 h after irradiation. Images represent ≈400 mm^2^ of coating surface. The percent change in hue angle of the 1:50, 1:100, and 1:150 Xa to TiO_2_ coatings with the C) larger and D) smaller TiO_2_ particles during the 72 h period after the sample was irradiated. The red dashed line indicates where the percent change in the hue angle is equal to zero. E) A cycling experiment where the 1:100 sample condition, both with larger and smaller TiO_2_ particles was irradiated and relaxed ten times to demonstrate that the coating could repeatedly switch between color states, without a significant change in the color of either state. For C–E) results are an average of three formulation replicates and error is reported as standard deviation.

We observed that after irradiating these samples, all of our coatings reverted back toward their original color in the absence of sunlight (**Figure** **2**A,B). To investigate i) if the coatings could fully recover their initial color and ii) how many times could the coatings cycle between yellow and red while maintaining their color, we irradiated the coatings for 30 min at 1100 W m^−2^, and then tracked color recovery over the course of 72 h. We calculated the percent change between the initial hue angle and the hue angle at a given timepoint to quantify color change and extent of recovery. For the 1:10 samples both prepared with larger and smaller TiO_2_ particles, we observed minimal initial change in hue angle and did not include these conditions in our color recovery experiments (Figure S7, Supporting Information). Overall, we observed that 50% of hue angle recovery occurred within the first 4 h of relaxation (Figure 2C,D) for all other conditions. The coatings recovered to 99% of their initial hue angle after 16.7 ± 15.0, 23.3 ± 24.8, and 30.7 ± 15.9 h for the 1:50, 1:100, and 1:150 samples with larger TiO_2_, and 26.7 ± 4.9, 39.7 ± 5.5, and 36.7 ± 9.2 h for the 1:50, 1:100 and 1:150 samples with smaller TiO_2_ particles, respectively. Overall, the samples with the larger TiO_2_ particles had a more inconsistent recovery time than the samples with the smaller TiO_2_ particles. This suggests that the smaller TiO_2_ not only offered more control over their final coating color as previously mentioned, but also demonstrated greater control of the color during the recovery after irradiation, as well as the rate of recovery. Interestingly, in most cases, the *a* and *b* color values that determine the hue angle of a sample were restored to the values of the original sample formulation within the 72 h timeframe. In some cases, the chromaticity value change during relaxation exceeded those of the original sample, which is why the final percent change in the hue angle for some samples was below zero. We suspect that this additional color change was due to the slight color change the polyurethane matrix undergoes when exposed to solar radiation that we previously observed (Table S1, Supporting Information).

To further characterize color changes in our samples, we calculated Δ*E* values (Equation (2)),^[^ ^40^ ^]^ to quantify the difference between colors of our coatings before initial irradiation and after relaxation and color recovery. Generally, when Δ*E* < 1, the differences in the color of two samples are undetectable to the human eye, and when 1 < Δ*E* < 2, the differences in color between two samples are only detected through close observation or by an experienced observer. Values above 2 indicate that a color difference between two samples is likely detectable to the average observer.^[^ ^41^ ^]^ Immediately after irradiation, the Δ*E* of the 1:50, 1:100, and 1:150 samples were 8.7 ± 0.5, 8.5 ± 0.3, and 8.5 ± 0.2 for the samples with the larger particles are 11.5 ± 0.3, 15.3 ± 1.4, and 16.1 ± 0.2 for the samples with the smaller particles; these results all indicate a visually obvious color change between the original sample and the sample directly after irradiation. At the end of the 72 h relaxation period, the Δ*E* values for the 1:50, 1:100, and 1:150 samples were 1.7 ± 0.1, 1.7 ± 0.5, and 1.8 ± 0.3 for the samples with larger TiO_2_ particles, and 1.3 ± 0.4, 1.6 ± 0.5, and 2.7 ± 1.0 for the samples prepared with smaller TiO_2_ particles (Figure S8, Supporting Information). Apart from the 1:150 sample with smaller TiO_2_ particles, these results further demonstrate the reversibility of the photochromic capabilities of these coatings, and that this behavior enables effective recovery to colors that are nearly indistinguishable from unirradiated samples. Changes in the coating color before and 72 h after irradiation may be more noticeable for the 1:150 sample with smaller TiO_2_ particles, but the results still demonstrate a high degree of color recovery with a Δ*E* change from 16.1 ± 2.1 to 2.7 ± 1.0.

Next, we explored how many times these coatings could transition between the oxidized and photoreduced forms without color degradation. To do this, we prepared samples with a 1:100 molar ratio of Xa to TiO_2_ using both sizes of TiO_2_ particles. We irradiated these samples using simulated solar light for 30 min and then stored them in the dark for 48 h (Figure 2E). For both formulations, we observed a smaller change in hue angle before and after the first irradiation compared to hue angle differences in later cycles. The hue angle changed by 2.5 ± 0.8° and 22.2 ± 1.3° on average in the initial irradiation cycle, but 7.2 ± 1.2° and 25.8 ± 2.2° on average in 9 subsequent irradiation cycles of the samples containing larger and smaller TiO_2_ particles, respectively. It is also interesting to note that for both conditions, after the initial irradiation, the oxidized form of the two coatings had hue angles that were 5.2 ± 2.3% and 7.6 ± 1.3% greater than the hue angle of the initial coating on average. This could potentially be attributed to a small amount of photobleaching at the surfaces of these paint films during initial irradiation events.

Overall, we determined that both conditions offer repeatable switching between the oxidized and reduced forms of Xa without meaningful color loss (Figure 2E; Figure S9, Supporting Information). For the 1:100 condition containing larger TiO_2_ particles, variation in the average hue angle over ten cycles was ± 1.7° for the oxidized form and ± 1.0° for the reduced form. Variation in the average hue angle for the oxidized and reduced forms were ± 1.4° and 1.9°, respectively, over 10 cycles for coatings containing smaller TiO_2_ particles. In electrochromic devices, Xa has offered reproducible color switching over more than 350 cycles,^[^ ^37^ ^]^ indicating that these coatings may be able to offer functional color switching over a long performance lifetime.

### 2.3 Investigating the Mechanism of Coating Photoreduction

**Figure 3:**
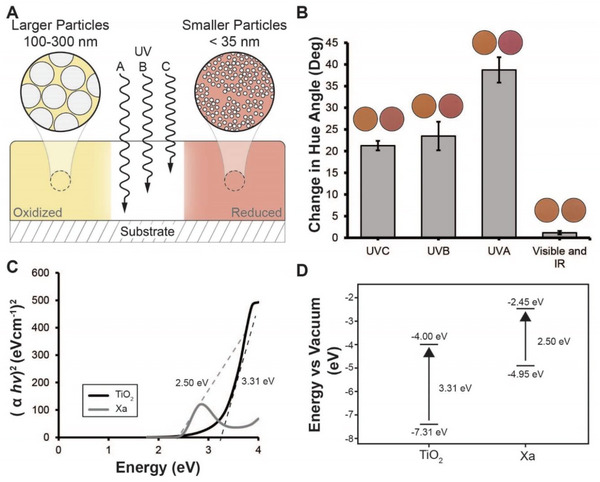
Energy dependence of photochromism in Xa‐based paints. A) Schematic depicting a cross‐section of the Xa‐TiO2 coatings. In these formulations, TiO_2_ particle size and loading density controlled the rate and intensity of Xa photoreduction. The different sized TiO_2_ particles are represented in the magnified part of the schematic and illustrate the differences in the accessible surface area between the two formulations. The arrows demonstrate that the polyurethane paint matrix differentially filters UVA, UVB, and UVC radiation through the depth of the coating (Figure S10, Supporting Information). B) Hue angle shift observed in 1:100 Xa:TiO_2_ formulations (smaller particles). Results include representative images before and after irradiation and hue angle values represent the average of three formulation replicates. Error is reported as standard deviation. C) Tauc plots for the direct energy bandgaps of TiO_2_ and Xa that were extrapolated from their UV–vis absorbance profiles. Dashed lines represent the tangent lines that were calculated to determine energy bandgaps. D) Comparison of the HOMO and LUMO positions of TiO_2_ and Xa at pH 7 and the corresponding direct energy bandgaps (see Supporting Information for calculations).

Our next goal was to deduce whether dynamic color change by our coatings was dependent on the energy of incident radiation. We prepared samples with a 1:100 molar ratio of Xa to TiO_2_ using the smaller particle size and exposed them to a consistent radiation dose (30 J cm^−2^) at 254 nm (UVC), 302 nm (UVB), 365 nm (UVA), and 400–1100 nm (vis–NIR) (**Figure** **3**A). Our results revealed that UVA radiation caused the greatest shift in hue angle while vis–NIR radiation generated the smallest color change (Figure 3B). The differences in hue angle before and after irradiation were 38.7 ± 2.9° and 1.2 ± 0.4° for UVA and vis–IR radiation, respectively. Samples irradiated with UVB and UVC radiation had hue angle changes of 23.5 ± 3.3° and 21.3 ± 1.1° and were not statistically different from each other.

Owing to the wide bandgap of TiO_2_, we observed minimal color change in coating samples that were irradiated with vis–NIR light. Both the anatase and rutile crystal forms of TiO_2_ require radiation of 400 nm or less to move electrons to an excited state.^[^ ^42^ ^]^ Therefore, the energy produced by the vis–NIR radiation was not sufficient to excite electrons in TiO_2_. As described previously, there is minimal change in the hue angle of coatings that do not contain TiO_2_ before and after irradiation, so this result further highlights that TiO_2_ plays a critical role in the color changing mechanism of our paints.

Because any radiation with a wavelength shorter than 400 nm is capable of exciting electrons in TiO_2_, it was interesting that UVA radiation generated a greater color change in our samples compared to UVB and UVC radiation (Figure 3B). To further evaluate this, we measured the transmission spectra of coatings prepared using only our polyurethane base material (Figure S10, Supporting Information). These results revealed that our polyurethane coating has low transmittance at representative wavelengths in the UVB and UVC regions, but readily transmits longer UVA wavelengths. It is possible that these energies were not able to penetrate the polyurethane matrix and only triggered Xa color change of the molecules at shorter penetration depths within the surfaces of our coatings. Because there is a significantly greater transmittance of UVA wavelengths through our coatings, these wavelengths likely penetrated deeper into our formulations to promote color change throughout the thickness of the sample as opposed to just on the surface, leading to intense color changes (Figure 3A,B).

To further explore the mechanism underlying Xa–TiO_2_ photochromism, we extrapolated estimated values for the energy bandgaps of both TiO_2_ and Xa. To do this, we measured the absorbance spectra of both materials (Figure S11, Supporting Information) and then converted the spectra to Tauc plots using Equation (3). We calculated estimates for the direct energy bandgaps of TiO_2_ and Xa to be 3.31 and 2.50 eV, respectively (Figure 3C; Figure S12, Supporting Information). Overall, the energy bandgap of TiO_2_ is greater, which supports other experimental findings that its absorption is limited to the UV portion of the solar spectrum. However, Xa has a smaller bandgap, supporting its broader absorption spectrum that spans the UV and visible regions (Figure 3D). Based on these calculations, we suspect that solar illumination may elicit differential electron injection between Xa and TiO_2_, resulting in redox‐based color change of our paint formulations. Future work will focus on continuing to investigate the mechanisms underlying light‐mediated photoreduction of Xa.

### 2.4 Alternative Colors and Patterning

**Figure 4:**
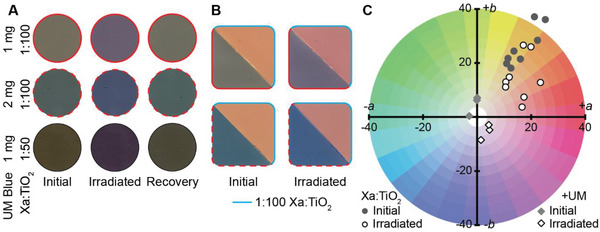
Expanding the color palette of Xa‐TiO_2_ coatings. A) Representative images of Xa coatings with supplemental Ultramarine blue before and after irradiation, as well as 24 h after irradiation. Images represent ≈400 mm^2^ of coating surface. B) Images of samples patterned with two different photochromic formulations that enable unique, simultaneous color changes. Images represent ≈500 mm^2^ of coating surface. C) CIE Lab color coordinate diagram depicting chromaticity values of Xa:TiO_2_ coatings with and without supplemental Ultramarine blue before and after irradiation.

Next, we highlighted potential applications of our coatings by increasing the range of accessible colors and performing selective irradiation to create transient geometric patterns of photoreduced Xa. Using varying concentrations and sizes of TiO_2_ particles in our formulation design, we established a color palette spanning yellows, tans, and reds (Figure 1). To further diversify the range of accessible colors, we decided to add supplementary, non‐photoresponsive natural pigments to blend against the dynamic color range of photoresponsive Xa‐TiO_2_ combinations (**Figure** **4**). We added Ultramarine, a commercially available form of the natural blue pigment lazurite, to the 1:50 and 1:100 Xa:TiO_2_ (smaller particle) coating formulations in 1 mg increments to produce three different shades of green. When irradiated, all samples shifted toward a purple hue (Figure 4A). Increasing the ratio of Ultramarine (UM) to TiO_2_ shifted blended colors toward blue tones, while the red color of photoreduced Xa blended with smaller quantities of UM to shift irradiated paints toward a deeper purple tone. All formulations shifted back toward their original color in the absence of sunlight. Additionally, we prepared individual samples composed of multiple color formulations that provided unique photochromic shifts, demonstrating more complex designs and increased hue density per area (Figure 4B). These new colors offered additional chromaticity range over our original formulations (Figure 4C). To further demonstrate this concept, we incorporated different quantities of soluble red dye to our original formulations, demonstrating that a variety of colorant types can be combined to diversify color using this approach (Figure S13, Supporting Information). Future work may focus on further expanding the color palette of our Xa‐based coatings, as well as exploring other color‐changing materials that could be paired with Xa or used in similar formulations.^[^ ^43^ ^]^

Additionally, we evaluated the performance of our coatings after exposure to simulated environmental conditions. We exposed samples of the 1:100 coatings with both the larger and smaller TiO_2_ particles to temperatures cycling between 30–43 °C over a 75 h period. There was a slight increase in the hue angle for both sample types after exposure, but the Δ*E* was 1.8 ± 0.7 and 1.4 ± 0.8 for the larger and smaller particles, respectively, indicating no significant change in color after the heat exposure (Table S3, Supporting Information). After 30 min of irradiation with simulated solar light, both types of coatings shifted to their photoreduced form, which led to prominent changes in the hue angle that were comparable to coatings with the same formulations that were not exposed to heat (Table S3 and Figure S14, Supporting Information**)**. For the samples prepared with larger TiO_2_ particles, the unexposed samples had an average hue angle shift of 9.7 ± 1.1° over 30 min of irradiation and the exposed samples had an average shift of 11.3 ± 0.4°. The unexposed sample with the smaller TiO_2_ particles had an average hue angle shift of 30.8 ± 2.5° and the exposed sample had an average shift of 26.7 ± 0.8°. During the 72 h color recovery period, samples with both TiO_2_ sizes recovered and then surpassed their original hue angle (Table S3, Supporting Information). The Δ*E* values after 72 h of relaxation for the larger and smaller TiO_2_ samples were 2.7 ± 0.1 and 2.8 ± 0.2, respectively. This suggests that the samples were able to mostly return to their original color, but there were small, almost undetectable differences in the color compared to the original sample color. These results provide some initial insight into the thermal stability of these coatings and their utility and performance outside of a laboratory environment. In future experiments, we may expand these environmental studies to other conditions, as well as examining and potentially improving the durability of the coatings via degradation studies.

**Figure 5:**
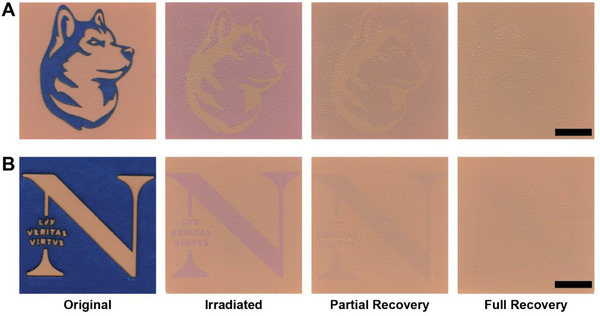
Selective irradiation of photoresponsive Xa‐TiO_2_ coatings. Geometric canine profile A) and typeface symbol B) patterned in masking tape and temporarily adhered to coating surfaces. After irradiation, the contrast of the reduced Xa pattern within the unirradiated surrounding substrate was distinctly visible and faded toward the original color of the substrate over the course of 72 h. Scale bars represent ≈12 mm.

Finally, we performed a proof‐of‐concept experiment to demonstrate that we could produce temporary complex geometric designs in these coatings using sunlight. To do this, we patterned adhesive tape using a CO_2_ laser to mask selected regions of coated surfaces from solar radiation, irradiating only selected areas to create visual designs (**Figure** **5**). This approach provided resolved patterns after irradiation, enabling visual identification of the intended design. Over time, these designs disappeared as photoreduced regions of the coating relaxed toward their original color, and these coated substrates could be used to create different geometric patterns. We imaged these patterns every hour for 72 h after irradiation to highlight how these complex designs faded over time (Movie S1, Supporting Information) to yield a blank, regenerated substrate. These results highlight how selective irradiation of our coating formulations enables rapid creation of reversible photochromic patterns, providing opportunities to create temporary designs and artwork on painted substrates.

## 3 Conclusion

We developed spray‐processable photochromic coatings composed of the natural biochrome Xa and the photocatalyst TiO_2_ that can reversibly shift between yellow (oxidized) and red (reduced) colors in response to solar radiation. In these systems, the presence and size of TiO_2_ was critical to the accessible color range and extent of color change, where the combination of these materials led to photoreduction in as little as 5 min of solar exposure. This process was reversible in the absence of sunlight, and color change could be induced at least ten times with no measurable loss of color or signs of pigment degradation. These results demonstrate the first step in our understanding of dynamic visible coatings enabled by natural colorants and highlight new opportunities to explore both the fundamental mechanisms that drive these performance capabilities, as well as applications for these materials in consumer goods or low‐power, optically active materials.

## 4 Experimental Section

### Xanthommatin Synthesis

We synthesized Xa via the oxidative cyclization of 3‐hydroxy kynurenine (3OHK) according to a previously published protocol.^[^ ^29^ ^]^ In summary, we first dissolved 3OHK (8 mg) in sodium hydroxide (1 mL, 25 mm) and then added potassium ferricyanide (33 mg dissolved in 1 mL of water) dropwise to the solution under stirring. We covered the solution to protect it from light and let it stir for 90 min at room temperature. Next, we added hydrochloric acid (1 mL, 1 m) dropwise and stirred the solution for 5 min to precipitate the Xa. Finally, we washed the precipitate via centrifugation three times with cold water and stored the material in a 4 °C refrigerator.

### Preparation and Application of Xa Coatings

The paint formulations consisted of a 1:10, 1:50, 1:100, 1:150, 1:200, or 1:250 molar ratio of Xa to TiO_2_ dissolved or suspended in a water‐based polyurethane matrix (Rust‐Oleum 6711). We tested two different sizes of TiO_2_ particles in our experiments. To prepare the samples, we first separately mixed the Xa (1 mg) and TiO_2_ (0.0019, 0.0094, 0.0189, 0.0283, 0.0378, or 0.0473 g) into the polyurethane paint (0.5 g) and then combined the two solutions (1 g total). We then sonicated each sample for 10 min and vortexed for 1 min, and repeated this process three times to produce samples of uniform consistency.

To apply the paint in an even manner, we prepared a mask made from painter's tape with a 1 × 1 inch square cutout and attached it to a piece of glossy cardstock. We applied the paint sample (0.8 g) to the substrate with an airbrush (Model G233, Master Airbrush). It was critical to vigorously vortex the sample before applying the paint to ensure that all the TiO_2_ was fully suspended. The samples dried for at least 12 h before irradiation or analysis.

### Irradiation of Xa Coatings

To examine the change in the paint color from irradiation over time, we first used a flatbed photo scanner (Epson Perfection V39) to obtain a high‐resolution image (600 dpi) of the original sample. We obtained two images every time a sample was imagined. One image was taken with an auto‐exposure feature activated and the other was with no image modifications. The images in this paper are the auto‐exposed images, but all the data was collected from the original, unmodified images. After imaging, we irradiated each sample using an arc lamp solar simulator (MKS Instruments, Newport Corporation) at a power density of 1100 W m^−2^ for 60 min. We removed the samples every 5 min and collected a high‐resolution image to track the color change of each sample. We reported our results as an average of three replicates and the error as ± one standard deviation.

### Color Analysis

To quantify the color changes in the samples due to irradiation, we measured values in the CIELab color space using ImageJ. In this color space, the *L* value represents the brightness of the sample and ranges from 0 (black) to 100 (white). The *a* value represents red (positive values) to green (negative values) chromaticity and the *b* value represents yellow (positive values) to blue (negative values) chromaticity. We used these values to calculate the hue angle (Equation (1)) and Δ*E* value (Equation (2)) of each sample. In Equation (1), *h°* is the hue angle, and *a* and *b* are the CIELab chromaticity values. In Equation (2), Δ*E* is the change in color between the initial and current sample, and Δ*L*, Δ*a*, and Δ*b* are the difference between the *L*, *a*, and *b* values of the original and current sample.

(1) $$h^{\circ }=\tan^{-1}\left(\frac{b}{a}\right)$$

(2) $$\Delta E=\sqrt{\Delta L^{2}+\Delta a^{2}+\Delta b^{2}}$$

### Color Recovery of Xa Coatings

To determine how long it takes for the samples to revert to their original color after irradiation, we scanned the original sample and then irradiated it with the solar simulator for 30 min at 1100 W m^−2^. We then obtained an image of the irradiated sample every hour for 72 h and presented our results as an average of three replicate samples and error as ± one standard deviation.

### Color Change of Coatings with Ultraviolet Radiation

For this set of experiments, we chose to use our formulation that consisted of a 1:100 molar ratio of Xa to TiO_2_ with the smaller particle size. We scanned our samples with a flatbed photo scanner prior to irradiation. To irradiate the samples, we used a handheld UV lamp (Analytik Jena) that emitted light at 254 nm (UVC), 302 nm (UVB), or 365 nm (UVA) and controlled the dose of irradiation that the samples were exposed to by adjusting the distance between the sample and the lamp to calibrate the irradiance. We used a UVA/B radiometer (SPER Scientific, 290–370 nm spectral range) to adjust the irradiance to 3.5 and 6.0 mW cm^−2^ in the UVA and UVB regions, respectively, and a UVC radiometer (General Tools, 220–275 nm spectral range) to set the irradiance to 3.5 mW cm^−2^ in the UVC region. All samples were exposed to 30 J cm^−2^ of irradiation. We imaged the samples with the photo scanner after irradiation and presented our results were an average of three replicates and error as ± one standard deviation.

### Color Change of Coatings with Visible/IR Radiation

In addition to ultraviolet irradiation, we wanted to determine the extent of color change in our coatings when they were exposed to solely to visible/IR radiation (400–1100 nm). We applied a UV filter film that blocked ≈90% of light under 390 nm onto the arc lamp solar simulator to isolate the visible/IR region. To achieve an energy dosage of 30 J cm^−2^, we calibrated the lamp to 100 mW cm^−2^, and irradiated the samples (1:100 molar ratio of Xa to TiO_2_ with the smaller particles) for 5 min. We captured a high‐resolution image of each sample before and after irradiation and presented our results as the average of three independent samples and error as ± one standard deviation.

### Statistical Analysis

All statistical analysis was done with a one‐way ANOVA test in Microsoft Excel. We set the significance threshold (p‐value) to 0.05. If any results were statistically significant from each other (*F* > *F_crit_*), we performed a Tukey test to determine which values were different from each other. In this test, we also set the significance threshold to 0.05.

### Energy Bandgap Calculations

To determine the energy bandgaps of TiO_2_ and Xa, we generated Tauc plots from the absorbance profiles with Equation (3). The first variable, *α* is the absorption coefficient and is determined by Equation (4). Next, *h* is Planck's constant (*h* = 6.62×10^−34^ Js), *v* is the frequency of incident light (*v* = *c*/λ, where *c* is the speed of light and λ is the wavelength), *A* is the optical constant, and *E*_g_ is the bandgap energy (*E*_g_ = 1240/λ). Lastly, *n* determines the type of bandgap, where *n* = 1/2 for a direct bandgap calculation, and *n* = 2 for an indirect bandgap calculation.

(3) $${\left(\alpha hv\right)}^{\frac{1}{n}}=A\left(hv-E_{g}\right)$$

(4) $$\alpha =\left(2.303\times \mathrm{Absorbance}\right)$$

To produce the Tauc plots, we plotted the bandgap energy (*E*_g_) as the *x*‐axis and (*αhv*)^1/n^ as the *y*‐axis for both direct and indirect bandgaps. For both the TiO_2_ and Xa, the direct bandgap equation was a better fit for the data. We drew a tangent line over the linear portion of the plot in Origin, and calculated the x‐intercept. This value is the energy bandgap of the materials.

### Development of Alternative Coating Colors

We expanded the color palate of our paint formulations by incorporating additional colorants to alter both the initial and irradiated color of the sample. To shift the color of the formulations from tan to green, we added 1 and 2 mg of ultramarine blue to the 1:100 Xa to TiO_2_ formulation and 1 mg of ultramarine blue to the 1:50 Xa to TiO_2_ formulation. We used the smaller TiO_2_ particles in these formulations. We applied these samples in the manner previously described and irradiated them for 30 min at 1100 W m^−2^. We imaged the samples before irradiation, after irradiation, and 24 h after irradiation to demonstrate that the coatings could return to their initial colors.

### Production of Temporary Patterns on Coating Surfaces

We created templatesto selectively control which parts of the coating surface were exposed to sunlight. To prepare these masks, we pressed three layers of painter's tape together to create a thick coating that prevented any light from going through the tape and discoloring the covered portions of the surface. We used Adobe Illustrator to create the designs and cut the designs into the adhesive with a CO_2_ laser system. We placed the masks on a coating prepared with a 1:100 molar ratio of Xa to the smaller TiO_2_ particles and irradiated for 30 min at 1100 W m^−2^. Next, we peeled the adhesive‐backed mask off of the coating to reveal the irradiated design. We then scanned the sample every hour for 72 h to demonstrate that the pattern was temporary and disappeared over time.

## Conflict of Interest

The authors declare no conflict of interest.

## Supporting information

Supporting Information

Supplemental Movie 1

## Data Availability

The data that support the findings of this study are available from the corresponding author upon reasonable request.
