# Supplementary material for: Color‐Changing Paints Enabled by Photoresponsive Combinations of Bio‐Inspired Colorants and Semiconductors
Source: Adv Sci (Weinh). 2023 Oct 3;10(32):2302652. doi: 10.1002/advs.202302652 (PMC10646264; doi:10.1002/advs.202302652)
Supplement: Supplementary file 1 — Supporting Information [file ADVS-10-2302652-s001.pdf]

ADVANCED SCIENCE
Open Access

Supporting Information

for *Adv. Sci.*, DOI 10.1002/advs.202302652

Color-Changing Paints Enabled by Photoresponsive Combinations of Bio-Inspired Colorants and Semiconductors

*Cassandra L. Martin, Kaitlyn R. Flynn, Taehwan Kim, Skyler K. Nikolic, Leila F. Deravi and Daniel J. Wilson**

Color-changing Paints Enabled by Photoresponsive Combinations of Bio-inspired Colorants and Semiconductors

Supplemental Materials

Cassandra L. Martin,[1] Kaitlyn R. Flynn,[1] Taehwan Kim,[2] Skyler K. Nikolic,[1] Leila F. Deravi,[2] & Daniel J. Wilson[1,3]*

*Corresponding author: da.wilson@northeastern.edu

[1]Kostas Research Institute at Northeastern University, Burlington, MA, 01803

[2]Department of Chemistry and Chemical Biology, Northeastern University, Boston, MA 02115

[3]Department of Chemical Engineering, Northeastern University, Boston, MA, 02115

1. Methods

1.1 pH Measurements

We prepared six different coating formulations in the water-based polyurethane at the 1 g scale: (1) the polyurethane alone, (2) the larger TiO_2 particles at the 1:150 molar ratio amount, (3) the smaller TiO_2 particles at the 1:150 molar ratio amount, (4) the Xa at the standard amount in all formulations, (5) the 1:150 coating formulation with the larger TiO_2 particles and (6) the 1:150 coating formulation with the smaller TiO_2 particles. Each sample was then painted onto a strip of pH paper and then the excess sample was wiped off. The pH strips were then imaged with a scanner.

1.2 Scanning Electron Microscopy (SEM) Imaging

We suspended the larger and smaller TiO_2 particles in distilled water and pipetted the sample (20 μL) onto a silicon wafer (Ted Pella Inc.) and let the water completely evaporate. We then imaged the samples with a scanning electron microscope (Scios 2 DualBeam, ThermoFisher Scientific).

1.3 Transmission Electron Microscopy (TEM) Imaging

We suspended both the larger and smaller TiO_2 particles in ethanol (70% v/v) and added a drop of each solution onto a carbon film Cu grid. We imaged the samples with a transmission electron microscope (Titan Thermis 300 S/TEM, ThermoFisher Scientific).

1.4 TiO_2 Particle Size Measurement

We used the SEM images to measure the particle sizes of both TiO_2 samples in ImageJ. We measured 50 particles for each condition. The results are presented as the average and the error is the standard deviation.

1.5 TiO_2 Surface Area Calculations

We approximated the surface area of the TiO_2 in each of our formulations using the average diameters of the larger and smaller TiO_2 we measured on the SEM. In addition, we assumed that the TiO_2 particles were spheres.

To calculate the surface area of the TiO_2 in the coatings we first calculated the volume of single particle (**Equation S1**), and then the mass of an individual TiO_2 particle using the known density of TiO_2 (4.23 g/cm^3). Since we applied 80% of our formulation onto the glossy cardstock, we

calculated the amount of TiO_2 that was in the coating that was applied, and then used the mass of a single TiO_2 particle to determine the amount of particles in the coating (**Equation S2**). We then calculated the surface area of a single TiO_2 particle assuming a spherical shape (**Equation S3**) and then multiplied that by the number of TiO_2 particles in the coating to get the final approximated surface area of the TiO_2 in each coating (**Equation S4**).

Equation S1: $Volume = \frac{4}{3}\pi r^3$

Equation S2: $Number\ of\ particles = \frac{Mass\ of\ particles\ in\ coating}{Mass\ of\ one\ particle}$

Equation S3: $Surface\ Area\ (SA) = 4\pi r^2$

Equation S4: $SA\ of\ TiO_2\ in\ coating = SA\ of\ one\ particle \times Number\ of\ particles$

1.6 Transmittance Spectrum Measurement of the Polyurethane Matrix

We applied the polyurethane matrix base paint on a piece of glossy cardstock over a 25.4 x 25.4 mm surface with an airbrush. After the sample dried, we peeled the coating off the cardstock with the tape to create a transparent thin film. We then measured the transmittance of the sample with an Ocean Optics Flame spectrophotometer.

1.7 Absorbance Spectrum Measurements

For the absorbance profiles for the Tauc plots, we diluted synthesized Xa and TiO_2 paste (Aqua Solution Inc.) in distilled water and added the samples to a 3 mL cuvette. We collected the absorbance profiles with a UV-Vis spectrophotometer (SpectaMax M5 series, Molecular Devices) from 200-700 nm.

1.8 Cyclic-Voltammetry Measurements

To determine the energy region of highest occupied molecular level (HOMO) and lowest unoccupied molecular level (LUMO), we conducted cyclic voltammetry (CV) with a three electrode system of glassy carbon (GC) electrode for working, silver-silver chloride (Ag/AgCl) in saturated potassium chloride (KCl) as reference, and Platinum (Pt) wire for counter electrode. We prepared the electrolyte by adding synthesized Xa (0.25 mg mL^{-1}) into phosphate buffered saline (0.1 M) and then adjusted the pH to 7. We measured the redox potential between a potential range of - 0.2 to 0.6 V with scanning rate of 100 mV s^{-1}. To determine the HOMO and LUMO region,

we used the following equations to extrapolate the oxidation and reduction peaks related to electron and hole injection into the conduction and valence bands.[1] In these equations, E_g was calculated based on the UV-Vis absorption spectra and Tauc plots (**Figure S11 and Figure 3C** respectively).

$$E_{NHE} = E_{Ag/AgCl} + 0.197$$

$$E_{HOMO} = -e(E_{oxidation\ vs\ NHE} + 4.75)\ (eV)$$

$$E_{LUMO} = -e(E_{reduction\ vs\ NHE} + 4.75)\ (eV)$$

$$or\ E_{LUMO} = E_g + E_{HOMO}$$

1.9 Development of Different Coating Colors with Red Dye 40

To further expand the color palette of our coatings, we incorporated a common red colorant called Red Dye 40 to our formulations. For our first set of experiments, we prepared the formulation with the 1:50 molar ratio of Xa to TiO_2 with the smaller TiO_2 particles (Method 4.2) and added Red Dye 40 in water (1, 2.5, and 5 µL, 0.5% w/w). In the next experiments we prepared both the 1:100 and 1:150 Xa to TiO_2 (smaller particles) formulations and added Red Dye 40 (10 and 2.5 µL, respectively). We applied the samples in the manner previously described and irradiated the samples for 30 minutes at 1100 W m^{-2}. We imaged the samples before and after irradiation and measured their LAB values with ImageJ.

1.10 Environmental Testing

We prepared three 1:100 samples with the larger TiO_2 particle and three 1:100 samples with the smaller TiO_2 particles. All samples were 25.4 x 25.4 mm and applied to a piece of glossy cardstock and imaged as explained in the Methods of the main manuscript. The samples were then placed in an environmental chamber (HD 205, Associated Environmental Systems) and run through the "Basic Hot" cycle in accordance with the MIL-STD-810H standard laboratory test.[2] After the cycle was complete, the samples were imaged again and then irradiated with the solar simulator at 1100 W m^{-2} for 30 minutes and then imaged again. The samples were then imaged 24, 48, and 72 hours after the irradiation to measure the color recovery.

2. Results

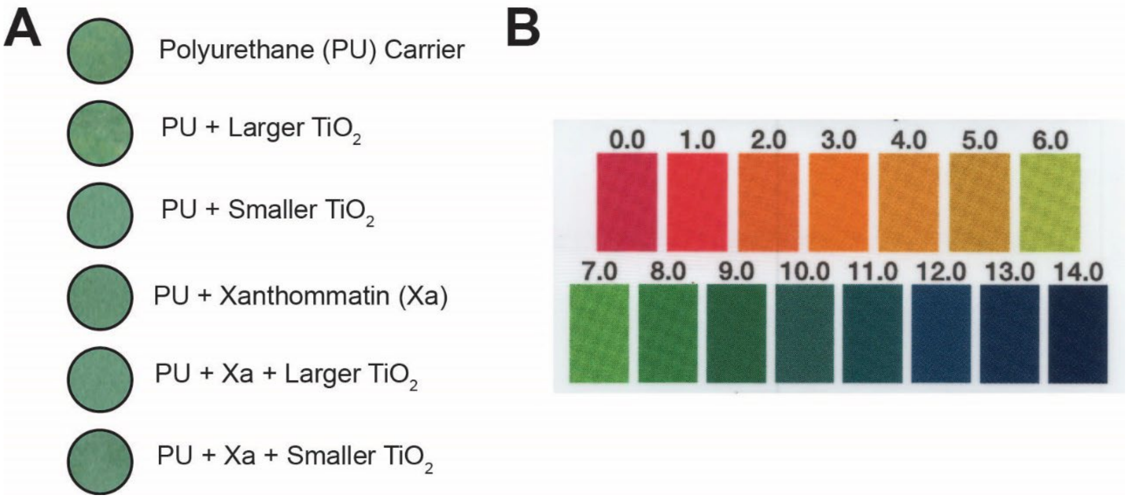

Figure S1. Analysis of the chemical environment of the coatings. (A) The pH of the water-based polyurethane with and without the Xa and TiO_2. (B) The key for the pH paper measurements.

Table S1. The hue angle of the coatings without TiO_2 before and after 60 minutes of irradiation.

Sample	Hue Angle Before Irradiation	Hue Angle After Irradiation	Change in Hue Angle
Polyurethane	-83.3 ± 0.2°	-81.6 ± 0.5 °	2.0 ± 0.5 %
Polyurethane and Xa	63.5 ± 0.6°	65.2 ± 0.4°	2.7 ± 0.4%

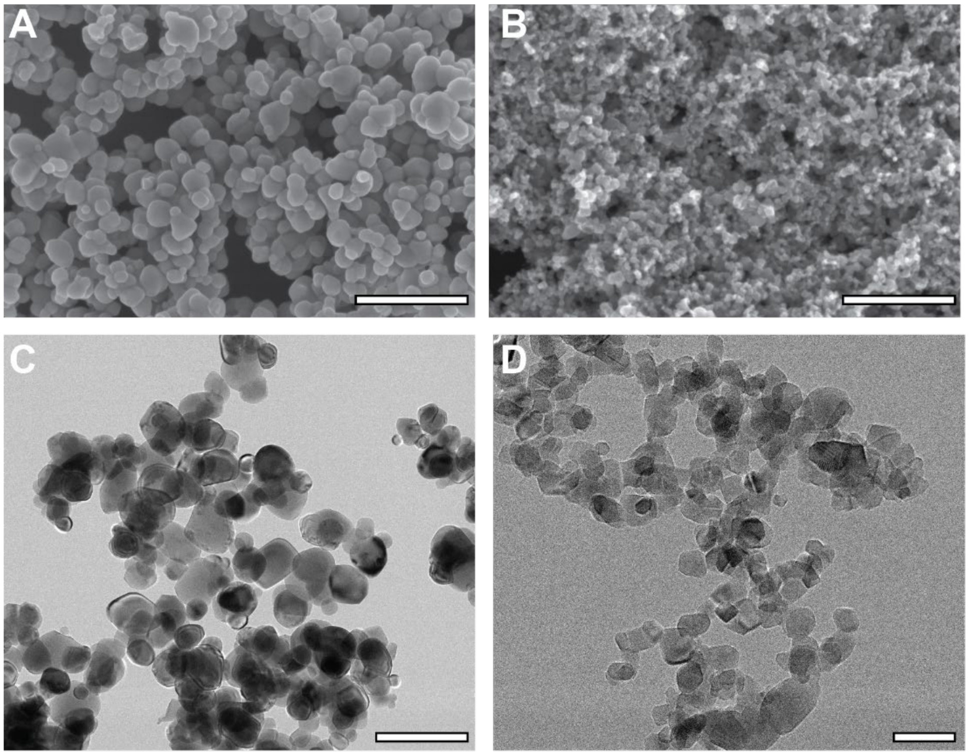

Figure S2. Particle Size of TiO_2. Representative SEM images of the (A) larger and (B) smaller TiO_2 used in the coating formulations. Scale bars are 1 μm and 500 nm, respectively. Representative TEM images of the (C) larger and (D) smaller TiO_2 particles. Scale bars are 500 and 50 nm, respectively.

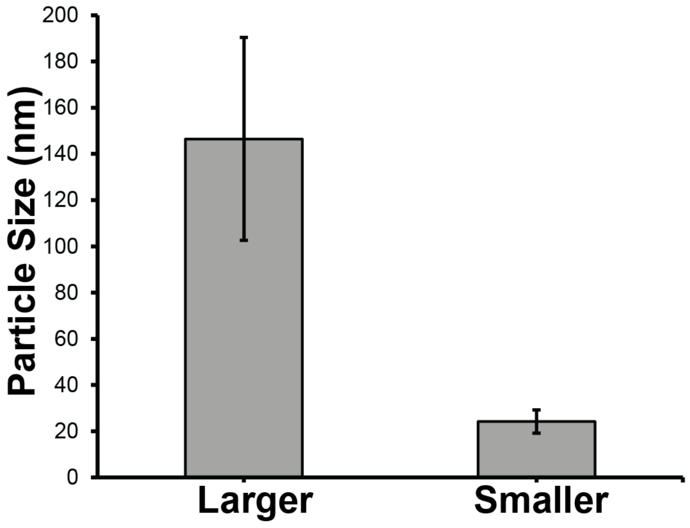

Figure S3. The average particle size for the larger and smaller TiO_2 particles. Results are an average of 50 measurements and error is reported as standard deviation.

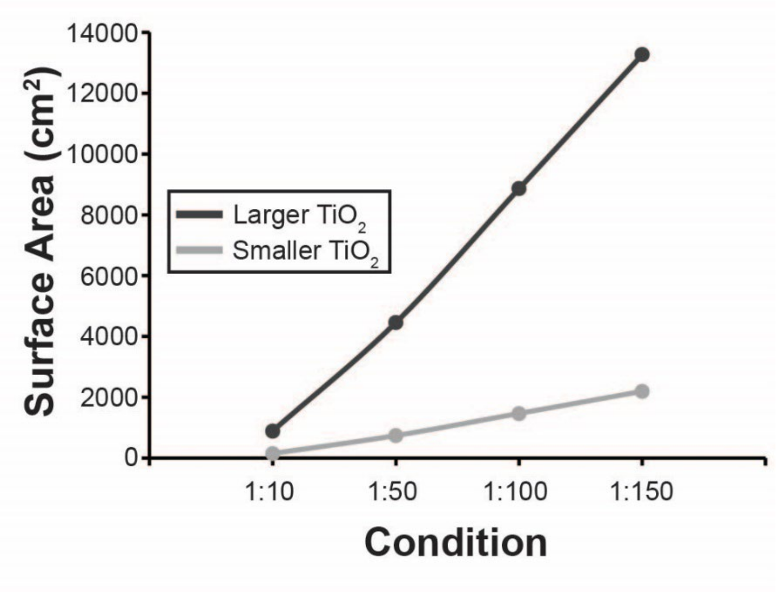

Figure S4. The surface area for the larger and smaller TiO_2 particles in the different coating formulations. Calculations were based off the average diameter measurements for the TiO_2 particles that were measured and reported in **Figures S2** and **S3**. Calculations were also based off the assumption that the TiO_2 particles were spheres.

Table S2. The brightness value (L) of each coating formulation before and after 60 minutes of irradiation.

Condition	TiO_2 Particle Size	Brightness (L)*
TiO_2 Before Irradiation**	Larger	95.7 ± 0.1
TiO_2 After Irradiation**	Larger	96.2 ± 0.1
TiO_2 Before Irradiation**	Smaller	92.3 ± 0.5
TiO_2 After Irradiation**	Smaller	92.8 ± 0.4
Xa Before Irradiation	N/A	62.6 ± 0.4
Xa After Irradiation	N/A	59.1 ± 0.5
1:10 Before Irradiation	Larger	59.1 ± 0.1
1:10 After Irradiation	Larger	55.6 ± 0.3
1:10 Before Irradiation	Smaller	54.0 ± 1.2
1:10 After Irradiation	Smaller	49.4 ± 0.8
1:50 Before Irradiation	Larger	66.3 ± 0.6
1:50 After Irradiation	Larger	63.6 ± 0.7
1:50 Before Irradiation	Smaller	58.9 ± 1.8
1:50 After Irradiation	Smaller	53.6 ± 2.3
1:100 Before Irradiation	Larger	70.3 ± 1.2
1:100 After Irradiation	Larger	67.3 ± 1.7
1:100 Before Irradiation	Smaller	63.3 ± 0.6
1:100 After Irradiation	Smaller	58.3 ± 0.2
1:150 Before Irradiation	Larger	74.1 ± 1.2
1:150 After Irradiation	Larger	71.8 ± 1.1
1:150 Before Irradiation	Smaller	67.6 ± 1.9
1:150 After Irradiation	Smaller	63.7 ± 2.0

* Results are an average of three formulation replicates and error is presented as the standard deviation.

** The samples were prepared with the amount of TiO_2 that is present in the 1:150 Xa to TiO_2 coating formulations.

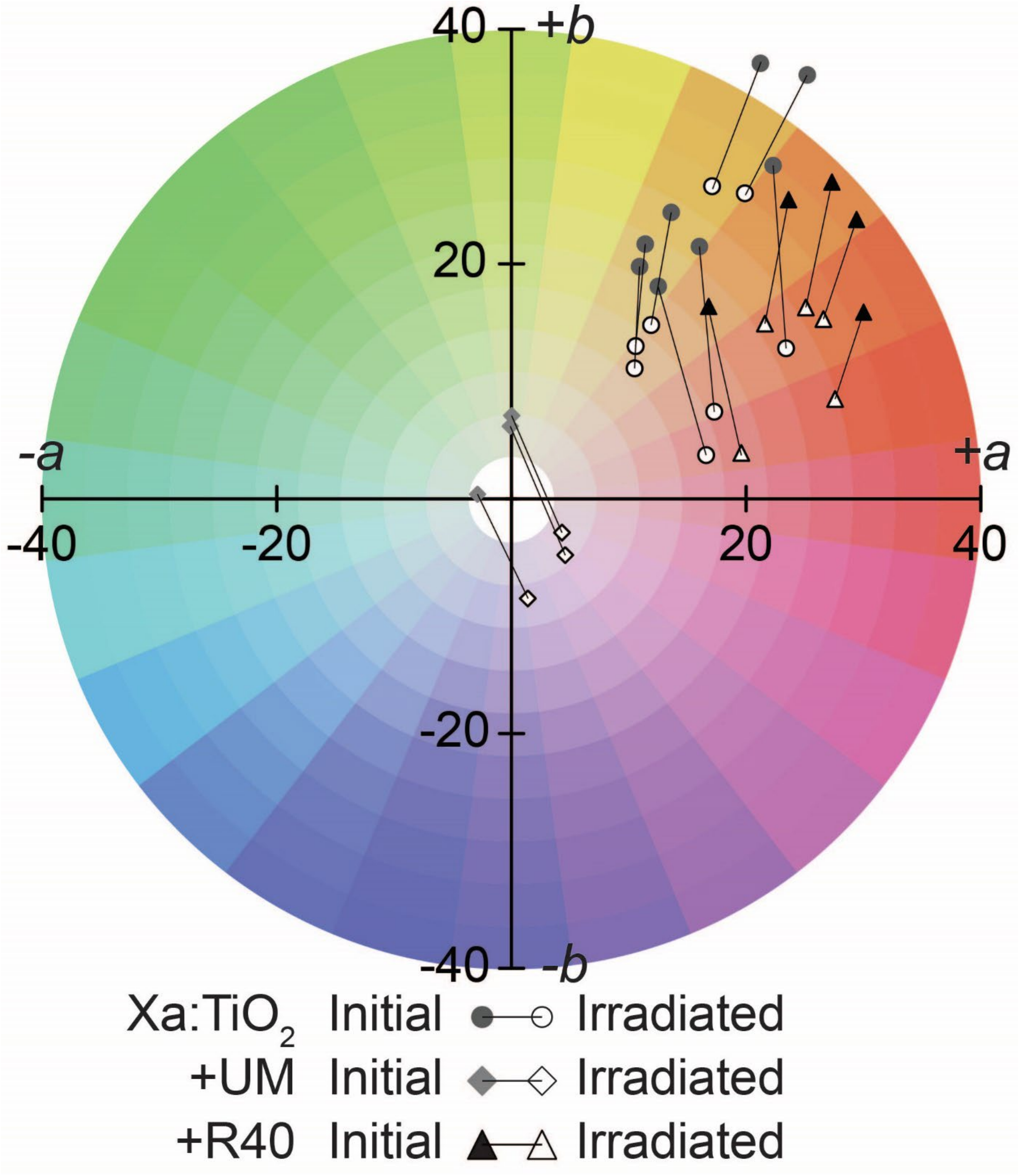

Figure S5. The CIELAB color coordinate diagram illustrating the *a* and *b* chromaticity values in our Xa-TiO_2 coatings. Points connected with a dotted line are the initial coating color and corresponding irradiated color of the same formulation.

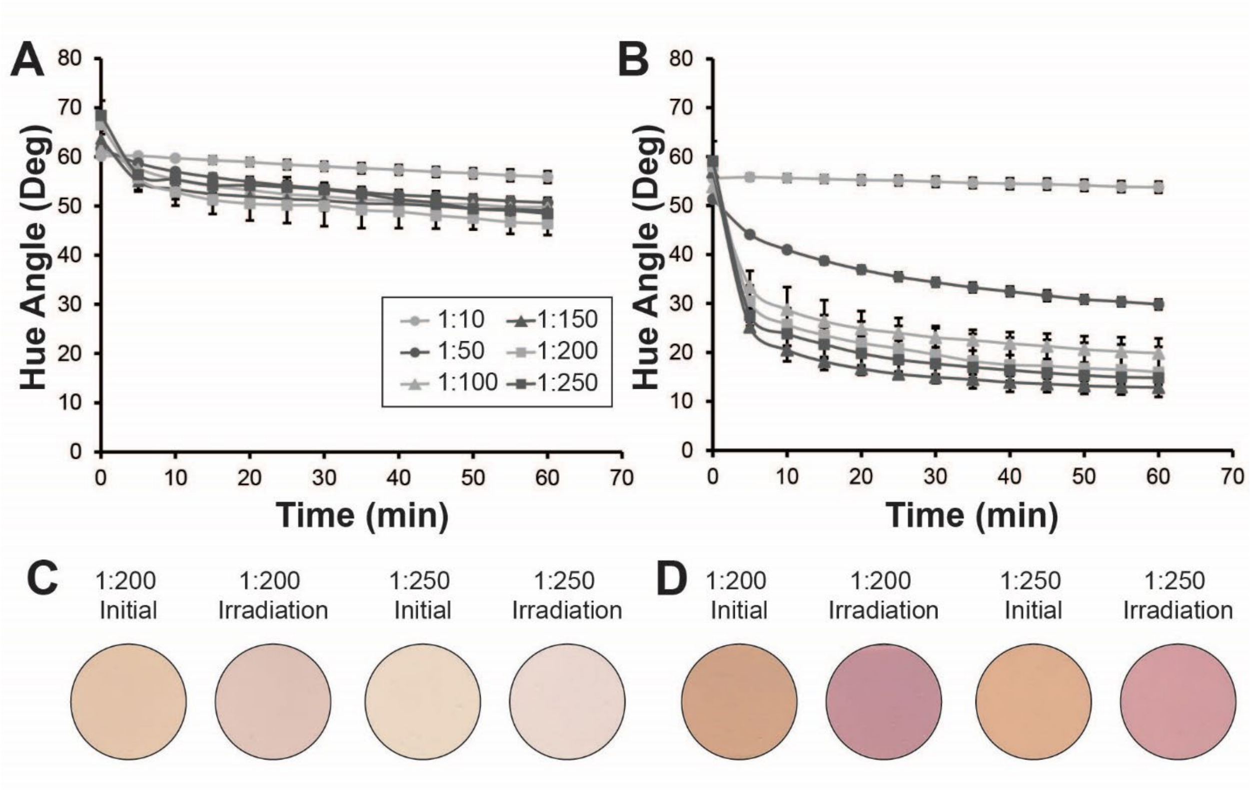

Figure S6. Photoreduction of Xa with higher TiO_2 concentrations. Change in hue angle upon irradiation over 60 minutes for coatings with a 1:200 and 1:250 molar ratio of Xa to TiO_2 with (A) larger TiO_2 particles and (B) smaller TiO_2 particles. Results are an average of three formulation replicates and error is reported as standard deviation. The other conditions in A and B are the other coating formulations that are in Figure 1 of the main text and are here so the color changing performance of all conditions can be compared. Representative images of the 1:200 and 1:250 coatings with (C) larger TiO_2 particles and (D) smaller TiO_2 particles before and after 60 minutes of irradiation. Images represent approximately 400 mm^2 of the coating surface.

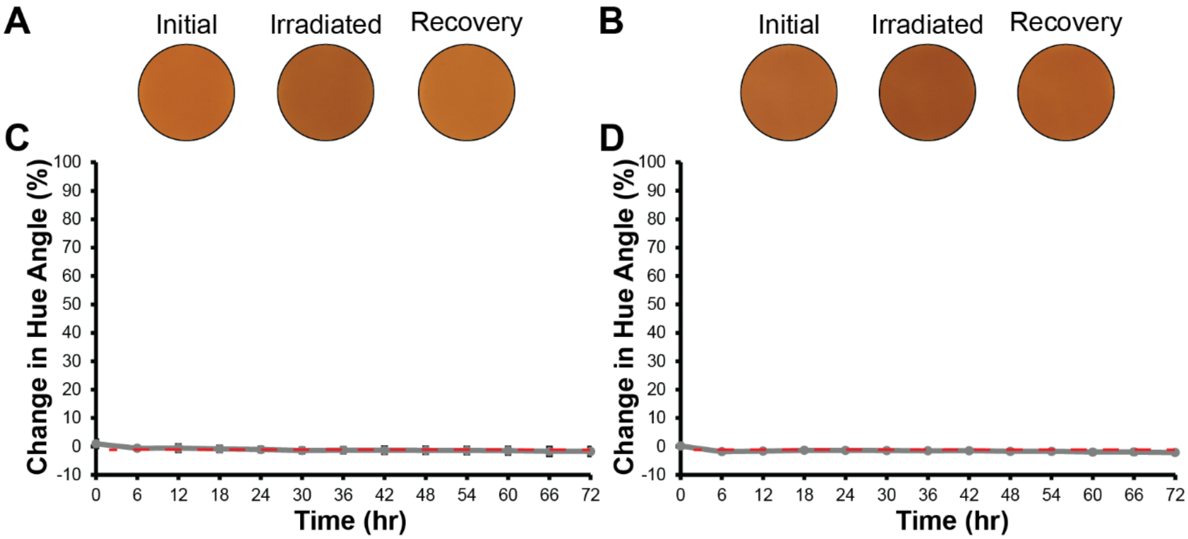

Figure S7. Color recovery of 1:10 samples. Representative images of the samples with a 1:10 molar ratio of Xa to (A) larger TiO_2 particles and (B) smaller TiO_2 particles before irradiation, after irradiation, and after a 72-hour relaxation period. (C) Results for the color recovery of the sample with the larger TiO_2 and (D) results for the color recovery of the sample with the smaller TiO_2 particles. Results are presented as the average of three formulation replicates and the error is reported as standard deviation. The red dashed lines mark where the change in hue angle is equal to 0%.

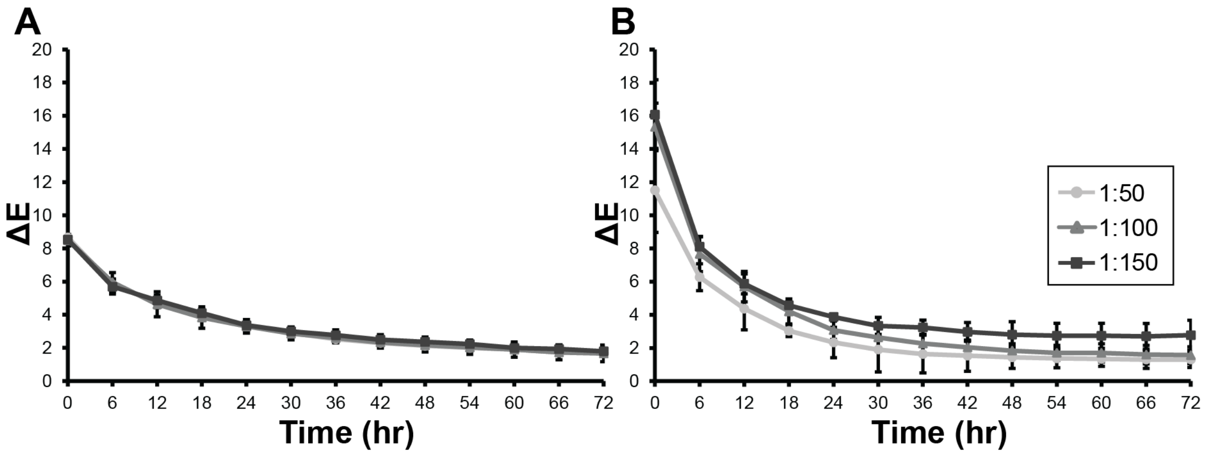

Figure S8. Change in ΔE over time during color recovery of coatings. We calculated the ΔE of each coating compared to the initial coating color immediately after (t=0) and over the next 72 hours. Results illustrated a decrease in ΔE over time for the samples with (A) larger and (B) smaller TiO_2 particles. Results are the average of three formulation replicates and the error is reported as standard deviation.

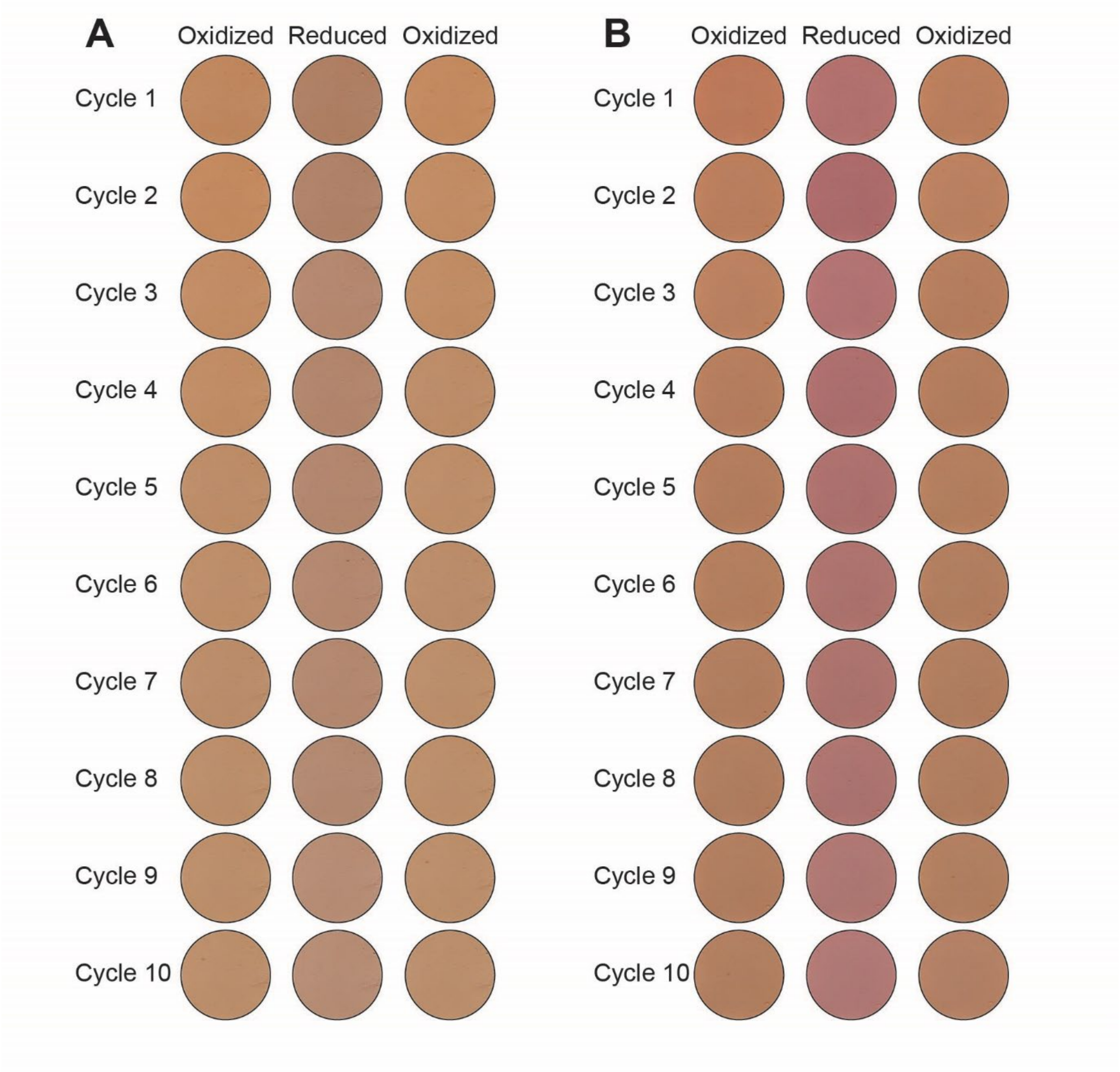

Figure S9. Cycling the coating color with sunlight. Representative images of the 1:100 coating formulation with (A) larger TiO_2 particles and (B) smaller TiO_2 particles.

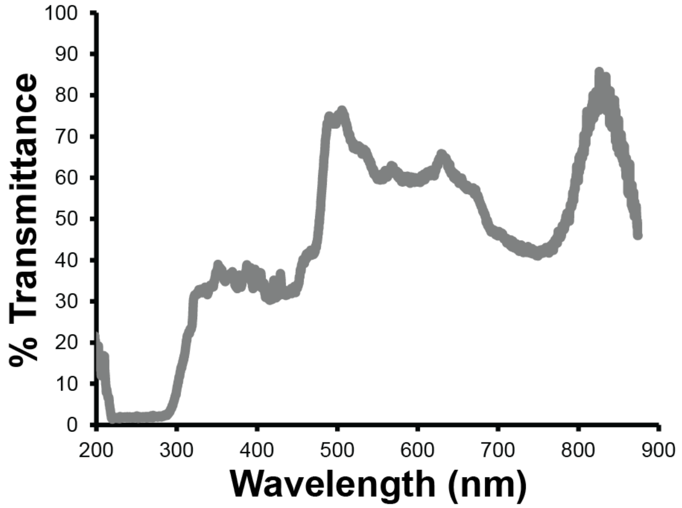

Figure S10. The transmittance profile of the polyurethane matrix from 200-900 nm.

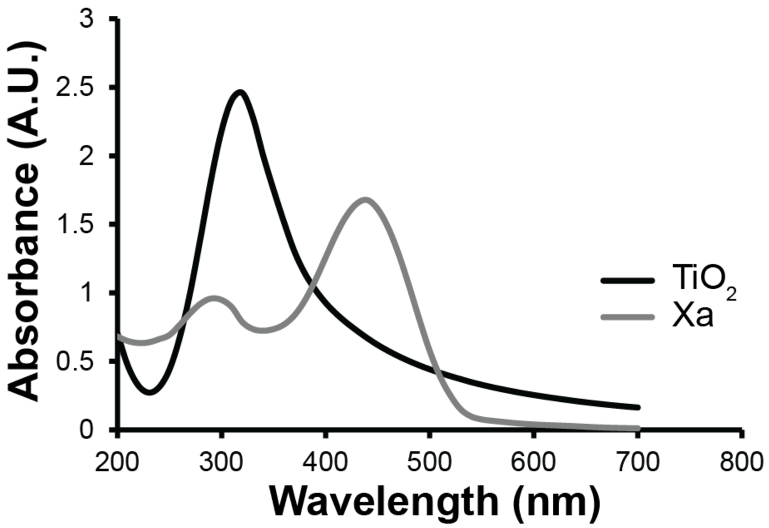

Figure S11. The absorbance spectra of TiO_2 and Xa in water from 200-700 nm.

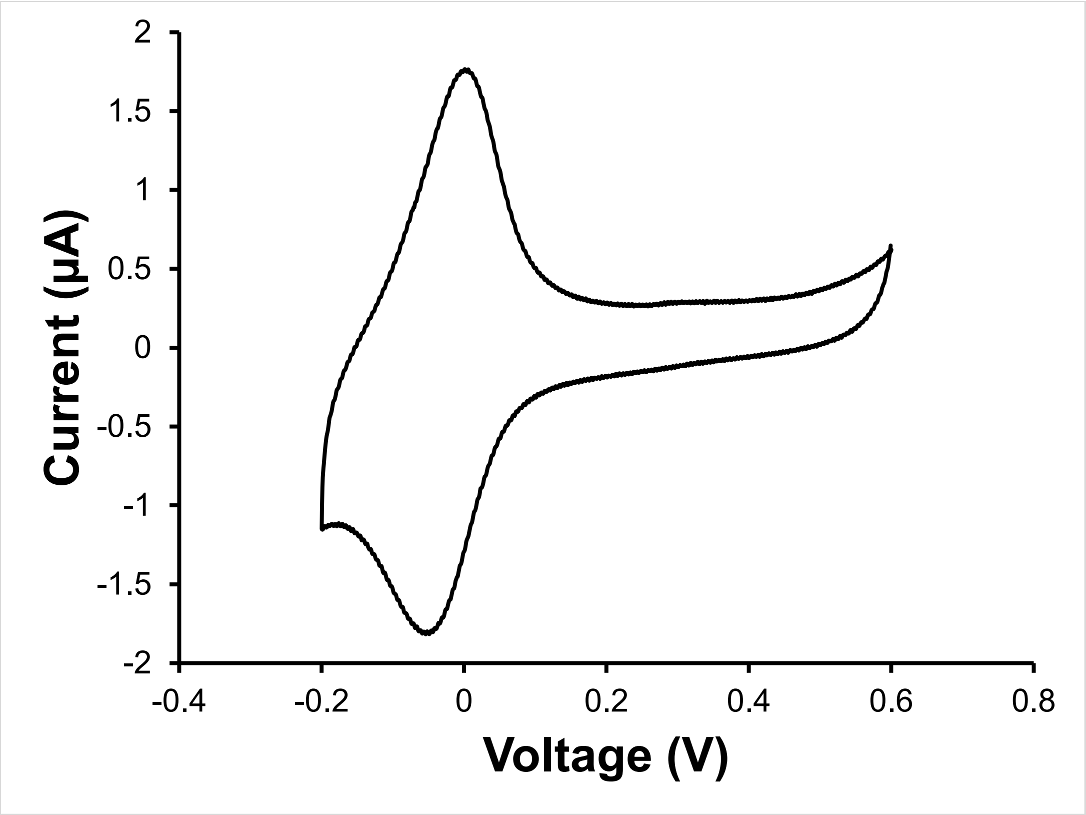

Figure S12. Cyclic-voltammetry diagram of Xa at pH 7 in 0.1 M PBS electrolyte.

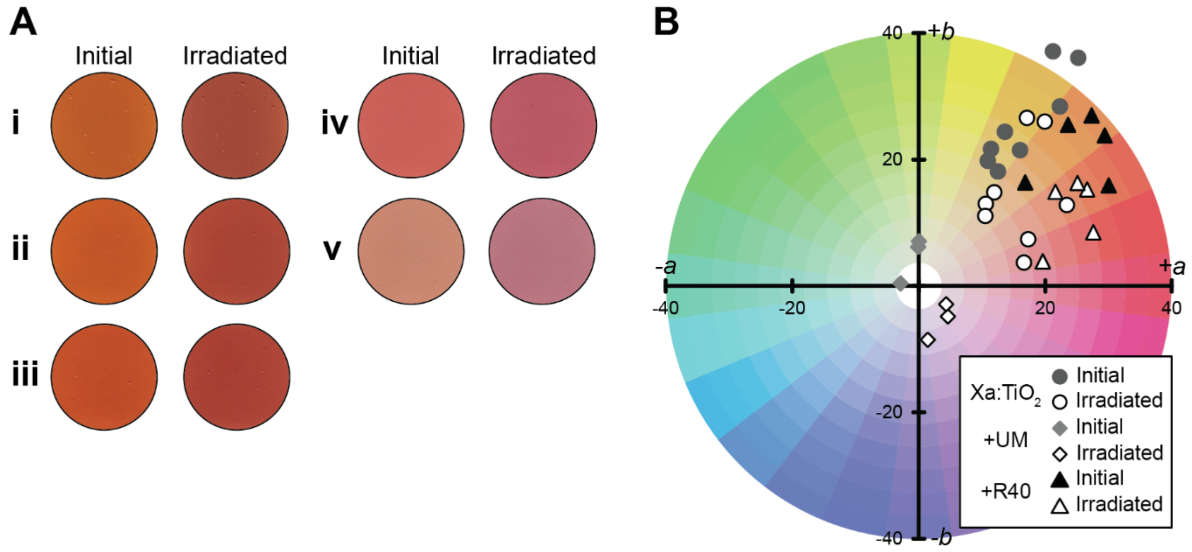

Figure S13. Addition of red colorant to coatings. (A) Representative images of coatings prepared with Red Dye 40 (R40) before and after 30 minutes of irradiation. The formulations are either the 1:50, (i, ii, iii) 1:100 (iv), 1:150 (v) molar ratio of Xa to smaller particle TiO_2 formulation with the addition of 1 (i), 2.5 (ii, v), 5 (iii), or 10 (iv) microliters of 0.5% (w/w) R40. Images represent approximately 400 mm^2 of coating surface. (B) CIELAB color coordinate diagram depicting chromaticity values of Xa-based coatings with supplemental non-responsive colorants before and after irradiation

Table S3[*]: The effect of the Basic Hot cycle on the Xa-TiO_2 coating function

Condition	Coating with Larger TiO_2 Hue angle (°)	Coating with Larger TiO_2 ΔE^{**}	Coating with Smaller TiO_2 Hue angle (°)	Coating with Smaller TiO_2 ΔE^{**}
Original	59.7 ± 0.3	N/A	53.5 ± 0.7	N/A
After Environmental Chamber	61.3 ± 0.1	1.8 ± 0.7	55.3 ± 1.3	1.4 ± 0.8
After Irradiation	49.9 ± 0.4	7.0 ± 0.1	28.6 ± 0.4	15.1 ± 0.3
24 hour Relaxation	64.0 ± 0.9	2.5 ± 0.2	54.8 ± 1.1	4.0 ± 0.6
48 hour Relaxation	62.5 ± 0.4	2.9 ± 0.3	56.5 ± 1.1	2.8 ± 0.2
72 hour Relaxation	64.5 ± 0.4	2.7 ± 0.1	56.7 ± 0.7	2.8 ± 0.2

* Results are an average of three formulation replicates and error is reported as the standard deviation.

** The ΔE values for the After Environmental Chamber condition compare the original sample color after application to the sample color after exposure in the environmental chamber. The ΔE values for the four conditions afterwards were calculated by comparing the given condition to the After Environmental Chamber condition.

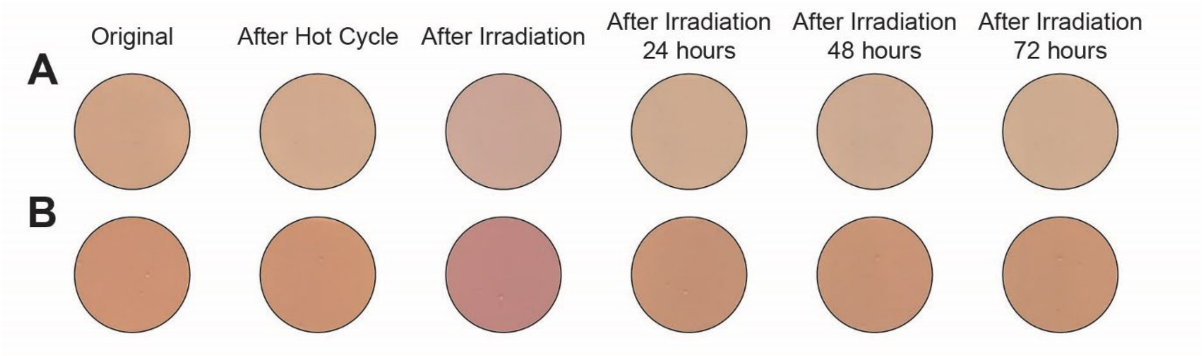

Figure S14. Performance of coatings after heat exposure. Representative images of the 1:100 coating formulations with the (A) larger TiO_2 particles and (B) smaller TiO_2 particles over the course of the Basic Hot Cycle environmental testing. After exposure in the environmental chamber, samples were exposed to the solar simulator for 30 minutes and then left to relax for 72 hours.

References:

[1] a) A. S.-I. Bayat, E., J Lumin 2017, 192 (180-183); b) L. Pan, B. Hu, X. Zhu, X. Chen, J. Shang, H. Tan, W. Xue, Y. Zhu, G. Liu, R.-W. Li, Journal of Materials Chemistry C 2013, 1 (30), 4556, https://doi.org/10.1039/C3TC30826J.

[2] MIL-STD-810H, Department of Defense Test Method Standard: Environmental Engineering Considerations And Laboratory Test 2019.
