# Supplementary figures and images for: Color‐Changing Paints Enabled by Photoresponsive Combinations of Bio‐Inspired Colorants and Semiconductors
Source: Adv Sci (Weinh). 2023 Oct 3;10(32):2302652. doi: 10.1002/advs.202302652 (PMC10646264; doi:10.1002/advs.202302652)

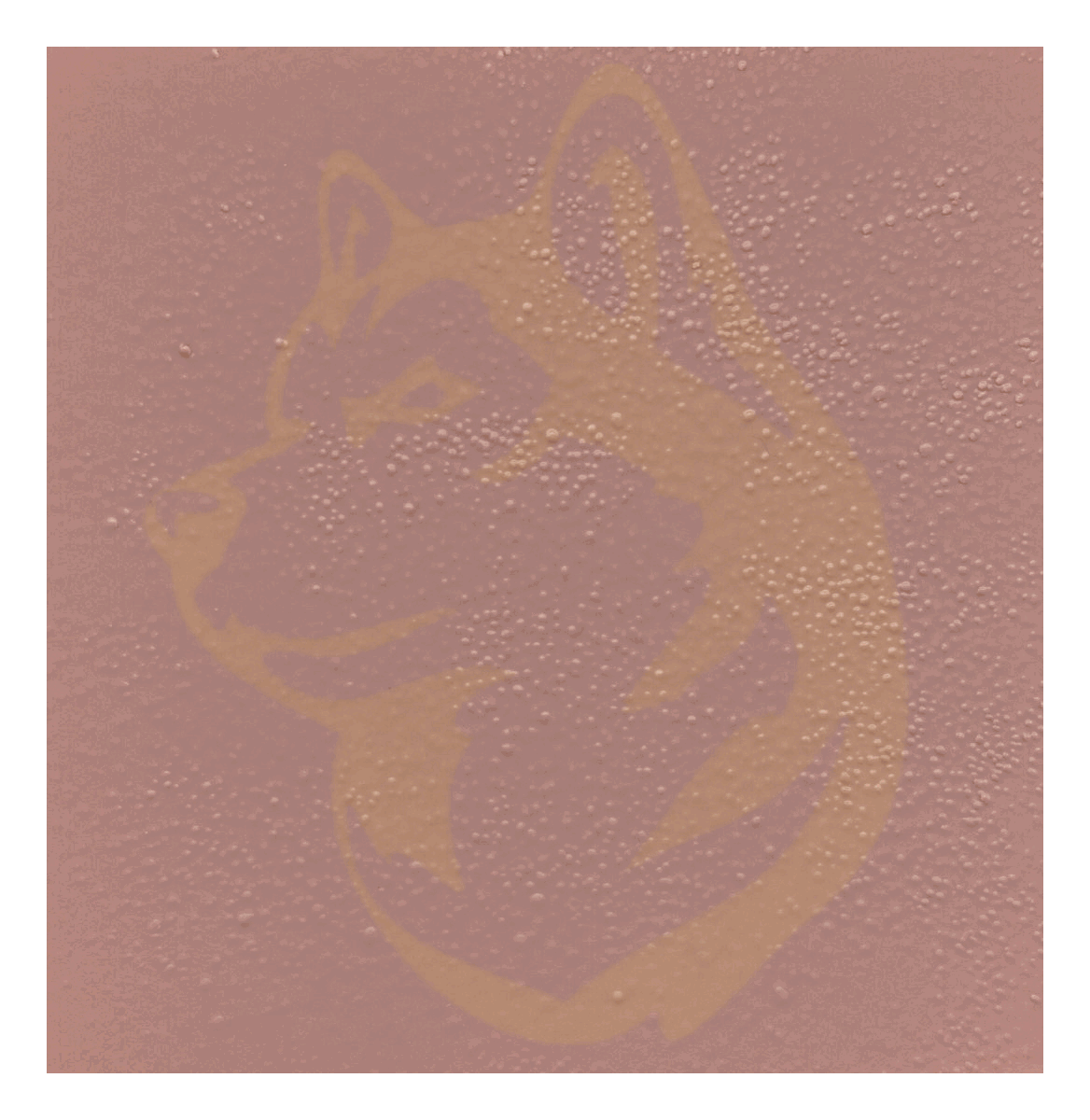

Supplement: Supplementary file 2 — Supplemental Movie 1 [file ADVS-10-2302652-s002.gif]
